# Reduced Hepatic Carcinoembryonic Antigen-Related Cell Adhesion Molecule 1 Level in Obesity

**DOI:** 10.3389/fendo.2017.00054

**Published:** 2017-03-27

**Authors:** Garrett Heinrich, Harrison T. Muturi, Khadijeh Rezaei, Qusai Y. Al-Share, Anthony M. DeAngelis, Thomas A. Bowman, Hilda E. Ghadieh, Simona S. Ghanem, Deqiang Zhang, Robert S. Garofalo, Lei Yin, Sonia M. Najjar

**Affiliations:** ^1^Department of Biomedical Sciences, Heritage College of Osteopathic Medicine, Ohio University, Athens, OH, USA; ^2^Diabetes Institute, Heritage College of Osteopathic Medicine, Ohio University, Athens, OH, USA; ^3^Center for Diabetes and Endocrine Research (CeDER), College of Medicine and Life Sciences, University of Toledo, Toledo, OH, USA; ^4^Department of Molecular and Integrative Physiology, University of Michigan Medical School, Ann Arbor, MI, USA; ^5^Yale Cancer Center, Office of Research Affairs, New Haven, CT, USA

**Keywords:** insulin clearance, insulin resistance, obesity, carcinoembryonic antigen-related cell adhesion molecule 1, hyperinsulinemia, fatty liver disease

## Abstract

Impairment of insulin clearance is being increasingly recognized as a critical step in the development of insulin resistance and metabolic disease. The carcinoembryonic antigen-related cell adhesion molecule 1 (CEACAM1) promotes insulin clearance. Null deletion or liver-specific inactivation of Ceacam1 in mice causes a defect in insulin clearance, insulin resistance, steatohepatitis, and visceral obesity. Immunohistological analysis revealed reduction of hepatic CEACAM1 in obese subjects with fatty liver disease. Thus, we aimed to determine whether this occurs at the hepatocyte level in response to systemic extrahepatic factors and whether this holds across species. Northern and Western blot analyses demonstrate that *CEACAM1* mRNA and protein levels are reduced in liver tissues of obese individuals compared to their lean age-matched counterparts. Furthermore, Western analysis reveals a comparable reduction of CEACAM1 protein in primary hepatocytes derived from the same obese subjects. Similar to humans, *Ceacam1* mRNA level, assessed by quantitative RT-PCR analysis, is significantly reduced in the livers of obese Zucker (*fa/fa*, ZDF) and Koletsky (*f/f*) rats relative to their age-matched lean counterparts. These studies demonstrate that the reduction of hepatic CEACAM1 in obesity occurs at the level of hepatocytes and identify the reduction of hepatic CEACAM1 as a common denominator of obesity across multiple species.

## Introduction

The carcinoembryonic antigen-related cell adhesion molecule 1 (CEACAM1) is ubiquitously expressed (1). CEACAM1 protein is expressed highly in liver, but to an insignificant extent in white adipose tissue and skeletal muscle, among classical insulin target peripheral tissues (1). Upon its phosphorylation by the insulin receptor tyrosine kinase in the hepatocyte (2), CEACAM1 promotes the uptake of insulin *via* its receptor to be degraded and cleared (3, 4). Bolstering this function of CEACAM1, defective hepatic insulin clearance and subsequently, chronic hyperinsulinemia develops in mice with global null mutation (*Cc1^−/−^*) or with liver-specific overexpression of the dominant-negative phosphorylation-defective inactive isoform of Ceacam1 (L-SACC1) (5–7). At least in part by downregulating the insulin receptor number (8), chronic hyperinsulinemia causes insulin resistance in these mice (5–7). Consistent with its positive effect on *de novo* lipogenesis (9), hyperinsulinemia also causes hepatic lipid accumulation, as well as lipid redistribution to the white adipose depot for storage, resulting in elevated visceral obesity. Contributing to visceral obesity and increased total fat mass in *Cc1^−/−^* mice is leptin resistance, manifested by hyperphagia and reduced spontaneous physical activity (10).

In humans and rodents, high-fat diet causes insulin resistance and visceral obesity. Recent data from our laboratories show that high-fat intake causes a decrease in hepatic CEACAM1 level by >50% within 3 weeks (11), and that this appears to play a causative role in diet-induced insulin resistance insofar as adenoviral-mediated delivery of CEACAM1 in liver reverses the metabolic abnormalities associated with increased fat intake, including insulin resistance, hepatosteatosis, and visceral obesity (12). Similarly, transgenic overexpression of CEACAM1 in liver protects against diet-induced insulin resistance, visceral obesity, hepatosteatosis, and fibrosis in adipose tissue (11).

Together, this assigns a significant role for reduced hepatic CEACAM1 levels in hyperinsulinemia-driven metabolic abnormalities, including insulin resistance and hepatic steatosis in mice. It also provided the impetus to investigate whether reduction of hepatic CEACAM1 level occurs at the hepatocyte level and whether it is common in obesity across multiple species.

## Materials and Methods

### Animal Care and Husbandry

Obese male Zucker fatty (*fa/fa*—8 weeks of age) and Zucker Diabetic Fatty rats (ZDF—12 weeks of age), and Koletsky spontaneous hypertensive rats (*f/f*—16 weeks of age) and their age-matched lean controls were purchased from Charles River Laboratories. Rats were fed *ad libitum* a regular chow diet and kept in a 12-h dark–light cycle. All procedures were approved by the Institutional Animal Care and Utilization Committee at the University of Toledo College of Medicine and Life Sciences (formerly known as the Medical College of Ohio). All experiments were conducted in accordance with the recommendations of the committee, confirming to the Guide for the Care and Use of Laboratory Animals published by the US National Institutes of Health (NIH Publication No. 85-23, revised 1996).

### Assessment of Plasma Biochemistry

Biochemical parameters were assessed in plasma drawn from overnight fasted rats. Plasma insulin and C-peptide levels were determined by radioimmunoassays (Linco Research) and their molar ratio at steady state was calculated as a marker of insulin clearance. Plasma triglyceride (TG) levels were assayed by Triglycerides reagent (Pointe Scientific) and plasma free fatty acids (FFA) by NEFA C kit (Wako). Hepatic TG content was assayed in tissues separated by chloroform–methanol, as previously described (12).

### Human Primary Hepatocytes

Livers and freshly isolated primary hepatocytes derived from the same lean and obese subjects were purchased from Cellzdirect (www.cellzdirect.com). The subjects include seven anonymous coded obese (body mass index >30 kg/m^2^) 45- to 50-year-old male subjects and four age-, sex-, and race-matched lean subjects. All subjects were non-smokers, non-alcoholics with no history of drug abuse, or other known health conditions or exposure to infectious diseases.

Specimens and cells were sent de-identified, labeled with a code with no other identifiable information. Hence, studies were exempted by the Institutional Review Board at the University of Toledo College of Medicine and Life Sciences (previously known as the Medical College of Ohio).

### Western Blot Analysis of Human CEACAM1 Protein Levels

Lysates from primary hepatocytes and liver were analyzed by 4–12% SDS-PAGE followed by immunoblotting (Ib) with polyclonal antibody against CEACAM1 (13), and normalization against GAPDH (Santa Cruz).

### Northern Blot Analysis of Rat *Ceacam1* mRNA Level

As previously described (11), Northern blot analysis was performed on total liver RNA extracted by TRIzol (Invitrogen), purified by MicroPoly (A) Pure Kit (Ambion), and sequentially probed with cDNAs for Ceacam1 followed by Gapdh for normalization, using the Random Primed DNA Labeling Kit (Roche).

### Quantitative RT-PCR Analysis of Rat *CEACAM1* mRNA Level

qRT-PCR was performed in homogenized liver lysates as routinely performed (14). Briefly, total RNA was extracted by TRIzol (GIBCO BRL) and first strand cDNA was synthesized using Superscript II (Invitrogen) and oligo dT, and real-time RT-PCR was carried out using the Applied BioSystem. The long isoform of *CEACAM1* was amplified using the following primers: F: 5′-CAGCGCTGGCATACTTCCTT-3′, R: 5′-CACTTCCCCCGCCAGTCT-3′. As control, *β-Actin* was amplified using the primers: F: 5′-ATCAAGATCATTGCTCCTCCTGA-3′, R: 5′GAGCCACCAATCCACACAGAG-3′. At least one primer of each pair is located in the junction of two exons to avoid amplification of genomic DNA. Ct values (cycle threshold) were used to calculate the amount of amplified PCR product relative to *β-Actin*. The relative amount of mRNA was calculated as 2^−ΔCT^.

### Statistical Analysis

Data were analyzed with SPSS software using one-factor ANOVA analysis or Student’s *t*-test. Values are expressed as mean ± SEM. ^A^*P* < 0.05 obese versus lean/genotype.

## Results

### Reduced Hepatic CEACAM1 Levels in Tissues from Obese Humans

**Figure 1:**
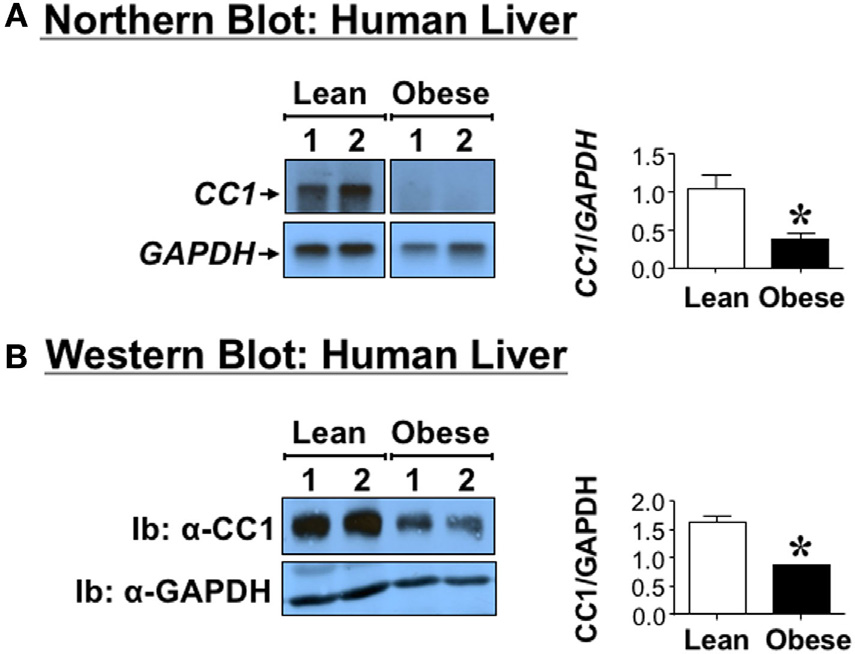
**Carcinoembryonic antigen-related cell adhesion molecule 1 (CEACAM1) level in human livers**. Livers derived from anonymous obese (body mass index >30 kg/m^2^) 45- to 50-year-old male subjects and age-, sex-, and race-matched lean subjects. **(A)** *CEACAM1* mRNA (*CC1*) was analyzed by Northern blot analysis of total liver mRNA and sequentially probed with cDNAs for *CEACAM1* (*CC1*) followed by *GAPDH* for normalization. **(B)** Liver lysates from obese and lean subjects were analyzed by 4–12% SDS-PAGE followed by immunoblotting (Ib) with polyclonal antibody against CEACAM1 (CC1) and normalization against GAPDH. For simplicity, only two samples of each group are shown as representatives of three independent experiments. The graph on the right represents densitometry analysis of CEACAM1 bands relative to those of GAPDH in all tissues. Values shown as mean ± SEM with *P < 0.05 being statistically significant.

Northern analysis indicates that *CEACAM1* mRNA levels, normalized to *GAPDH*, are significantly lower (by ~>60%) in the liver of obese human subjects by comparison to those derived from their lean sex-, race-, and age-matched counterparts (Figure 1A). This translates into reduced hepatic CEACAM1 protein levels in lysates derived from livers (Figure 1B) of obese human subjects, as assessed by Western blot analysis using Ib with antibodies against human CEACAM1 and GAPDH (to normalize against total protein loading). Moreover, obese subjects exhibit hepatic fat accumulation, as assessed by the twofold to threefold higher hepatic TG level in obese subjects (50.2 ± 4.5 versus 20.3 ± 2.2 mg/g liver tissue, *P* < 0.05).

### Reduced CEACAM1 Protein Content in Primary Hepatocytes from Obese Humans

**Figure 2:**
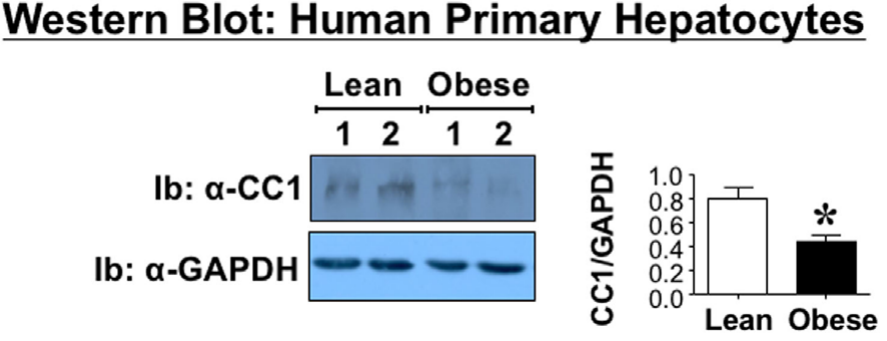
**Carcinoembryonic antigen-related cell adhesion molecule 1 (CEACAM1) level in human primary hepatocytes**. Primary cells were derived from the livers of the same anonymous donors and analyzed by Western analysis as in Figure 1, immunoblotting (Ib) with polyclonal antibody against CEACAM1 (CC1) and normalizing against GAPDH. For simplicity, only two samples of each group are shown as representatives of three independent experiments. The graph on the right represents densitometry analysis of CEACAM1 bands relative to those of GAPDH in all cells. Values shown as mean ± SEM with *P < 0.05 being statistically significant.

Because metabolic factors such as insulin and fatty acids regulate Ceacam1 expression in hepatocytes, with insulin inducing its transcription (15) and fatty acids repressing it (16), we then aimed to examine whether the decline in hepatic CEACAM1 occurs at the hepatocyte level. To this end, we examined the protein level of CEACAM1 in primary hepatocytes derived from the same obese and lean subjects whose livers were used to assess hepatic CEACAM1 levels (see above). Western blot analysis using antibodies against human CEACAM1 for Ib indicates ~50% reduction (graph) in CEACAM1 protein level in primary hepatocytes derived from obese as compared to their sex- and age-matched lean counterparts (Figure 2).

### Reduced Hepatic CEACAM1 Levels in Obese Rats

**Figure 3:**
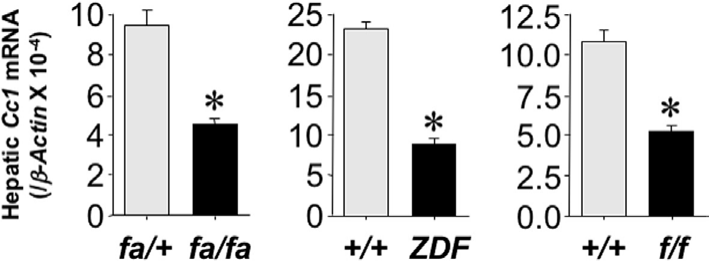
**Hepatic carcinoembryonic antigen-related cell adhesion molecule 1 (CEACAM1) level in obese rodents**. *Ceacam1* mRNA was analyzed by qRT-PCR analysis of total liver RNA, normalized to *β-Actin*, from livers derived from obese *fa/fa*, ZDF, and Koletsky *f/f* rats and age-matched lean controls (*n* = 10/lean or obese/each strain). Values shown as mean ± SEM with *P < 0.05 being statistically significant.

To investigate whether the reduction in hepatic CEACAM1 in obesity is common among species, we then examined mRNA levels of *Ceacam1* in the livers of obese male rats. These include obese Zucker hyperphagic rats without diabetes (*fa/fa*) or with diabetes (Zucker Diabetic Fatty rats—ZDF) (17), and obese spontaneous hypertensive Koletsky rats (*f/f*) (18). qRT-PCR analysis revealed a ≥50% decrease in hepatic *Ceacam1* mRNA levels in obese relative to lean rats (Figure 3). Consistent with a role for CEACAM1 in insulin clearance (6), obese rats display reduced insulin clearance (as measured by steady-state C-peptide/insulin molar ratio) and hyperinsulinemia (Table 1). As expected, they also exhibit elevated body weight, fasting plasma FFA, and plasma and hepatic TG levels (Table 1).

**Table 1:** **Biochemical parameters in obese rats**.

	Zucker *fa/fa*		Zucker ZDF		Koletsky (*f/f*)	
	Lean	Obese	Lean	Obese	Lean	Obese
Body weight (g)	254 ± 10	330 ± 9^A^	275 ± 11	352 ± 10^A^	315 ± 9	458 ± 12^A^
Insulin (ρM × 10^2^)	3.0 ± 0.8	18.2 ± 1.6^A^	0.9 ± 0.1	7.2 ± 1.0^A^	1.0 ± 0.3	25.5 ± 0.6^A^
C-peptide (ρM × 10^3^)	1.1 ± 0.2	3.3 ± 0.2^A^	1.3 ± 0.1	3.0 ± 0.4^A^	1.5 ± 0.4	5.2 ± 0.2^A^
C-peptide/insulin ratio	3.8 ± 0.6	1.8 ± 0.1^A^	15.7 ± 1.3	5.0 ± 0.5^A^	14.6 ± 0.6	2.0 ± 0.1^A^
FFA (mEq/l)	0.5 ± 0.0	1.4 ± 0.1^A^	0.6 ± 0.1	1.1 ± 0.2^A^	0.7 ± 0.1	1.1 ± 0.2^A^
TG (mg/dl)	14 ± 3	246 ± 42^A^	65 ± 3	531 ± 58^A^	112 ± 8	385 ± 29^A^
Hepatic TG (mg/g tissue)	23 ± 2	63 ± 7^A^	27 ± 3	73 ± 5^A^	34 ± 4	78 ± 9^A^

*Frozen liver tissues and plasma were extracted from 10 obese and 10 age-matched lean overnight fasted rats. These include fa/fa (8 weeks of age); ZDF (12 weeks of age), and Koletsky *f/f* (16 weeks of age). Values are expressed as mean ± SEM*.

*^A^P < 0.05 obese versus lean/genotype. C-peptide/insulin molar ratio was used as determinant of insulin clearance*.

*FFA, free fatty acids; TG, triglycerides*.

## Discussion

Using several genetically modified mouse models of loss- and gain-of-function of Ceacam1, we have demonstrated that CEACAM1 plays a critical role in promoting hepatic insulin clearance, and that its loss in the liver causes chronic hyperinsulinemia followed by systemic insulin resistance, altered lipid homeostasis, hepatosteatosis, and visceral obesity (5–7). That defective insulin clearance contributes significantly to these obesity-associated metabolic abnormalities has been demonstrated in several species, including humans (19–22). Thus, it has become imperative to investigate whether hepatic CEACAM1 level is commonly reduced among species. The current studies demonstrate that by comparison to lean controls, CEACAM1 level is reduced in the liver of age- and sex-matched obese human subjects and in three rat models of obesity resulting from null mutation of leptin receptor (17, 23).

Although the data on human subjects need to be strengthened by a much larger cohort of patients, they are consistent with a report finding a marked decline in hepatic CEACAM1 levels in 29% of 99 obese subjects with insulin resistance and non-alcoholic fatty liver disease, with a higher incidence of CEACAM1 loss in individuals with high-grade fatty liver and severe obesity, independently of type 2 diabetes (24). That this occurs independently of diabetes and fasting hyperglycemia is consistent with normal insulin secretion and fasting normoglycemia in Ceacam1 mutant mice (7). Moreover, sustained reduction of CEACAM1 protein content in primary hepatocytes derived from the same steatotic livers of obese donors demonstrates that the defect in CEACAM1 expression occurs at the hepatocyte level. We have recently shown that the rise in fatty acids release from adipocytes during high-fat feeding of mice progressively represses Ceacam1 expression in the hepatocyte by activating a mechanism depending on the activation of peroxisome proliferator-activated receptor α by fatty acids (25) and that this bestows a positive feedback mechanism on fatty acid β-oxidation (12). When the loss of hepatic CEACAM1 reaches more than 50% and impairment of insulin clearance develops, chronic hyperinsulinemia followed by hepatic steatosis ensues (12). Increased lipolysis-driven hepatic fatty acid β-oxidation in humans with uncomplicated obesity (26) and its role in regulating hepatic *de novo* lipogenesis (27, 28) propose an important role for the loss of hepatic CEACAM1 in the regulation of lipid homeostasis in hepatocytes derived from obese humans.

Obese Zucker and Koletsky hyperphagic obese rats display a decline in their hepatic CEACAM1 content likely causing impaired insulin clearance and hyperinsulinemia. They also manifest elevated visceral obesity with high fasting plasma FFA, and an increase in plasma and hepatic TG levels, consistent with the phenotype of *Ceacam1* mutant mice (5–7).

Similarly, rats selectively bred for low aerobic running capacity (LCR) exhibit metabolic syndrome, including hyperinsulinemia, insulin resistance, obesity, and hypertension. By comparison to age-matched high capacity runners (HCR) (29), they also exhibit hepatic steatosis (30). Hyperinsulinemia in LCR rats is associated with impaired hepatic insulin clearance in correlation with reduced *Ceacam1* mRNA (29) and protein levels (14). Caloric restriction reduces their hyperinsulinemia, and subsequently, hepatic fatty acid synthase level and steatosis, in parallel to inducing hepatic CEACAM1 levels and normalizing hepatic insulin extraction to the level of HCR (14). Whether low hepatic *Ceacam1* level in LCR by comparison to HCR is a cause or a consequence of increased release of plasma FFA from the white adipose tissue in these rats (12, 25) remains to be determined, but it is intriguing that CEACAM1 expression is modulated by the selection for low aerobic running capacity that leads to the metabolic anomalies of LCR rats.

In summary, the current studies demonstrate a strong association between reduced CEACAM1 expression in hepatocytes with obesity, hepatic steatosis, and dyslipidemia across species and multiple rat strains.

## Author Contributions

GH researched data, designed experiments, and wrote the manuscript. HM, HG, SG, KR, QA-S, AD, TB, and DZ researched data. RG designed experiments, and extracted tissues and plasma from rats. LY designed experiments in human tissues and reviewed the manuscript. SN was responsible for study design, conceptualization, data analysis, and results interpretation, and reviewing the manuscript; had full access to all the data of the study and takes responsibility for the integrity and accuracy of data analysis and the decision to submit and publish the manuscript.

## Conflict of Interest Statement

The authors declare that the research was conducted in the absence of any commercial or financial relationships that could be construed as a potential conflict of interest.
